# Epigenetic silencing of the XAF1 gene is mediated by the loss of CTCF binding

**DOI:** 10.1038/srep14838

**Published:** 2015-10-07

**Authors:** Georgina Victoria-Acosta, Karla Vazquez-Santillan, Luis Jimenez-Hernandez, Laura Muñoz-Galindo, Vilma Maldonado, Gustavo Ulises Martinez-Ruiz, Jorge Melendez-Zajgla

**Affiliations:** 1 Functional Cancer Genomics Laboratory, National Institute of Genomic Medicine, Mexico D.F., 14610, Mexico; 2 Epigenetics Laboratory, National Institute of Genomic Medicine, Mexico D.F., 14610, México; 3 Unit of Investigative Research on Oncological Disease, Children’s Hospital of Mexico “Federico Gomez”, Mexico City, Mexico

## Abstract

XAF1 is a tumour suppressor gene that compromises cell viability by modulating different cellular events such as mitosis, cell cycle progression and apoptosis. In cancer, the *XAF1* gene is commonly silenced by CpG-dinucleotide hypermethylation of its promoter. DNA demethylating agents induce transcriptional reactivation of XAF1, sensitizing cancer cells to therapy. The molecular mechanisms that mediate promoter CpG methylation have not been previously studied. Here, we demonstrate that CTCF interacts with the *XAF1* promoter *in vivo* in a methylation-sensitive manner. By transgene assays, we demonstrate that CTCF mediates the open-chromatin configuration of the *XAF1* promoter, inhibiting both CpG-dinucleotide methylation and repressive histone posttranslational modifications. In addition, the absence of CTCF in the *XAF1* promoter inhibits transcriptional activation induced by well-known apoptosis activators. We report for the first time that epigenetic silencing of the *XAF1* gene is a consequence of the loss of CTCF binding.

The tumour-suppressor gene X-linked inhibitor of the apoptosis (XIAP)-associated factor 1 (XAF) favours apoptosis by inhibiting XIAP^1^,^2^,^3^,^4^,^5^, which is one of the most important members of the inhibitors of apoptosis protein (IAP) family. In addition, XAF1 also presents XIAP-independent proapoptotic actions that contribute to its tumour suppressor gene activity^6^,^7^,^8^. XAF1 expression is absent or decreased in gastric^9^, ovarian^10^, pancreatic^11^, esophageal^12^, colon^9^, hepatic^13^, melanoma^14^ and urogenital tumours^15^,^16^,^17^. Although loss of heterozygosity has been showed to be associated to XAF1 expression absence^18^, promoter CpG dinucleotide hypermethylation appears to be the principal cause of altered XAF1 expression^9^,^12^,^17^. Exposure to demethylating agents such as 5-azacytidine readily induces the reestablishment of XAF1 expression, thereby increasing the sensitivity to drug-induced apoptosis^12^,^19^,^20^. In xenograft models, ectopic XAF1 expression impedes tumour formation and prolongs the survival of tumour-bearing mice^21^,^22^. However, the molecular mediators of the hypermethylated state and decreased expression are currently unknown.

CTCF is a multitask protein involved in gene regulation. This protein functions as a transcriptional regulator, enhancer blocker and chromatin barrier^23^. These actions are secondary to its main function as a genome-wide organizer of chromatin architecture^24^,^25^. The biological actions of CTCF are explained by its ability to function as a DNA-binding protein scaffold. CTCF interacts with its DNA-binding sites in a methylation-sensitive fashion, thereby impeding the methylation of imprinting control regions^26^,^27^. In cancer, it has been described that CTCF is able to modulate the histone posttranslational modification (HPM) status and CpG methylation from several tumour suppressor genes^28^.

Here, we demonstrated that CTCF directly regulates XAF1 expression by binding to a methylation-sensitive CTCF-binding site in its promoter. The absence of CTCF promotes epigenetic silencing of the *XAF1* promoter by both accelerated CpG-dinucleotide methylation and the transition from active to repressive HPMs. Importantly, in cancer cell lines, the lack of CTCF regulation on the *XAF1* promoter via methylation on its cognate binding site partially blocks its transcriptional responsiveness to two well-known transcriptional activators, TNF-α or IFN-α. These findings uncover for the first time an epigenetic mechanism involved in establishing the repressive configuration of the *XAF1* promoter and, consequently, transcriptional unresponsiveness.

## Results

### Specific CpG-dinucleotide methylation impedes full XAF1 responsiveness to either TNF-α or IFN-α in MCF-7 cells

**Figure 1:**
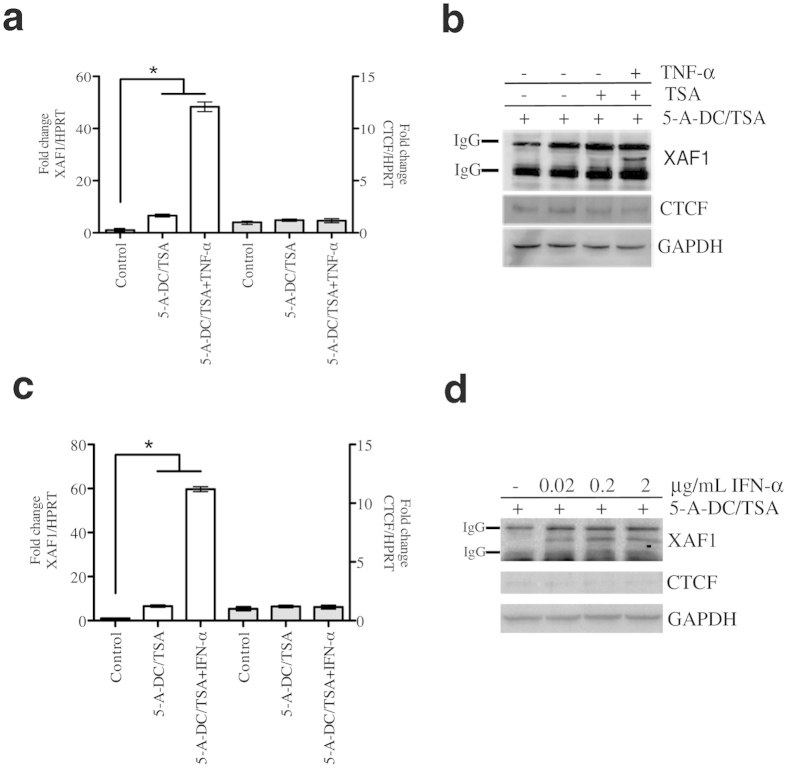
XAF1 expression is induced by either TNF-α or IFN-α in demethylating conditions. (**a**) MCF-7 cells were pre-treated with *5*-Aza*-*2′-deoxycytidine (5 μM) and Trichostatin-A (0.2 μM) for 3 days before stimulation with TNF-α (20 ng/mL) for 24 h. Quantitative PCR (qPCR) analysis of *XAF1* and *CTCF* mRNA expression was performed. *HPRT* mRNA was used as loading control. Results are presented in terms of fold change. The means from three independent experiments were plotted with ±SEM, *P < 0.05. (**b**) MCF-7 cells were treated as shown in (**a**). Using a specific antibody, XAF1 was immunoprecipitated from equal quantities of total extracted proteins for each condition. XAF1, CTCF and GAPDH protein levels were measured by Western blot. (**c**) MCF-7 cells were pre-treated as in (**a**) before stimulation with IFN-α at the indicated concentrations. mRNA expression of both *XAF1* and *CTCF* was analysed by qPCR after normalizing with *HPRT* mRNA. The mean from three independent replicates were plotted with +SEM, *P < 0.05. (**d**) MCF-7 cells were pre-treated and stimulated as shown in (**c**). Western blot analysis was performed as shown in (**b**). 5*-*Aza*-*2′-deoxycytidine (5-A-DC); Trichostatin-A (TSA).

**Figure 2:**
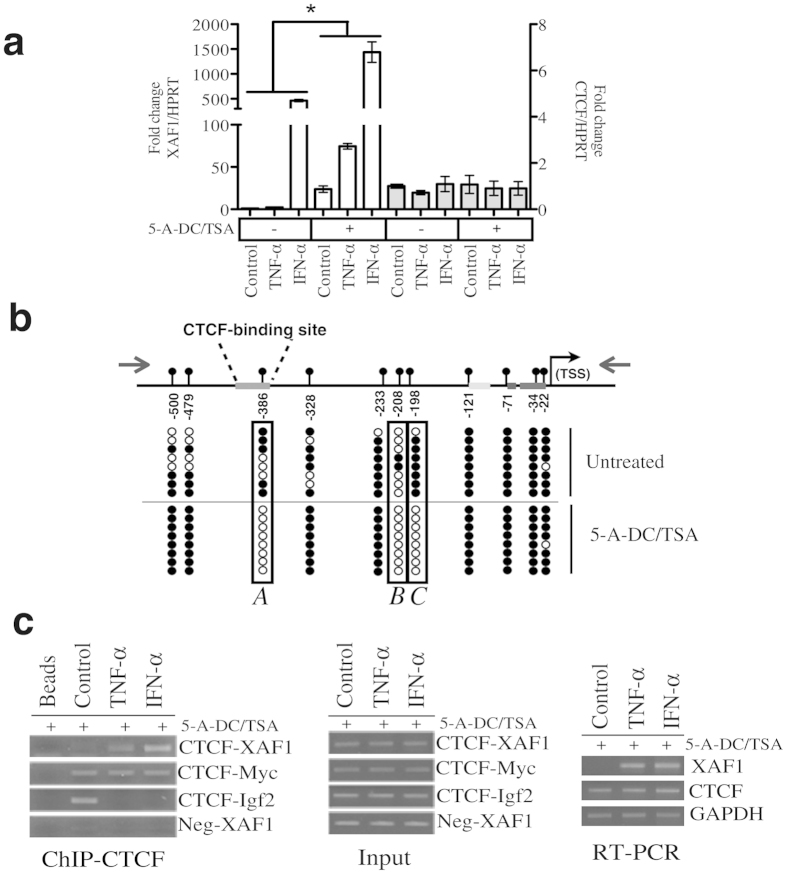
CTCF interacts with the *XAF1* promoter. (**a**) ACHN cells were pre-treated with 5*-*Aza*-*2′-deoxycytidine (5 μM) and Trichostatin-A (0.2 μM) for 3 days before stimulation with either TNF-α or IFN-α. Non-demethylated cells were stimulated with either TNF-α or IFN-α. mRNA expression of *XAF1*, *CTCF* and *HPRT* was analysed by qPCR. Results are presented in terms of fold change. The means from three independent experiments were plotted with ±SEM, *P < 0.05. (**b**) MCF-7 cells were either treated or not treated with demethylating agents, as shown in (**a**). Bisulphite sequencing was then performed. A schematic representation of the XAF1 promoter shows the locations of 11 CpG-dinucleotides sites from −22 to −500 bp relative to the TSS. Methylated and unmethylated CpGs are depicted as filled and open circles, respectively (**c**) MCF-7 cells were treated as shown in (**a**). ChIP assays were performed using a specific antibody against CTCF protein. The CTCF-binding site in the *XAF1* promoter was analysed by PCR in the DNA recovered after ChIP (Left panel). As positive and negative controls of CTCF-DNA interaction, three specific sets of primers were included. Two of them were directed to previously validated CTCF binding sites (c-Myc and IGF2) as positive controls, and one was a negative control. The input represents soluble chromatin that was reversed cross-linked and amplified by PCR (central panel). RT-PCR was performed from cells used for ChIP assays. 5*-*Aza*-*2′-deoxycytidine (5-A-DC); Trichostatin-A (TSA).

As expected based on previous reports showing that *XAF1* promoter is hypermethylated in cancer^9^,^12^,^17^, here, pre-exposure to demethylating agents increased the transcriptional activation of XAF1 in basal conditions (Supplementary Fig.1a). To test XAF1 dynamic expression, we used two well-known XAF1 transcriptional activators, TNF-α and IFN-α^29^,^30^,^31^ Demethylating conditions were required to display full transcriptional activation of XAF1 at both the mRNA and protein levels after TNF-α (Fig. 1a,b) or IFN-α (Fig. 1c,d) exposure. To extend these observations to another unrelated cancer cell line, we used ACHN cells, which have previously been shown to be responsive to IFN-α in demethylating conditions^32^. As observed with MCF-7 cells, we observed a dramatic increase in XAF1 responsiveness in demethylating conditions (Fig. 2a). As a positive control, we used the Colo205 cell line that presents an unmethylated XAF1 promoter^9^. Even without previous exposure to epigenetic modifiers, we observed a clear XAF1 transcriptional activation by TNF-α exposure (Supplementary Fig. 1b). We then reasoned that differential dinucleotide CpG methylation between control cells and cells treated with demethylating agents could help us to identify which DNA segments are important for the full responsiveness of XAF1. To this end, we performed bisulphite genomic sequencing using a specific set of primers to amplify the *XAF1* promoter. Exposure to 5-aza-2′-deoxycytidine (5-A-DC) and trichostatin-A (TSA) induced consistent demethylation of three CpG dinucleotides in MCF-7 cells (Fig. 2b; A, B and C). These results indicate that full transcriptional activation of the *XAF1* gene is associated with a specific CpG-dinucleotide methylation state of its promoter.

### CTCF interacts with the *XAF1* promoter when cells are stimulated with TNF-α or IFN-α

**Figure 3:**
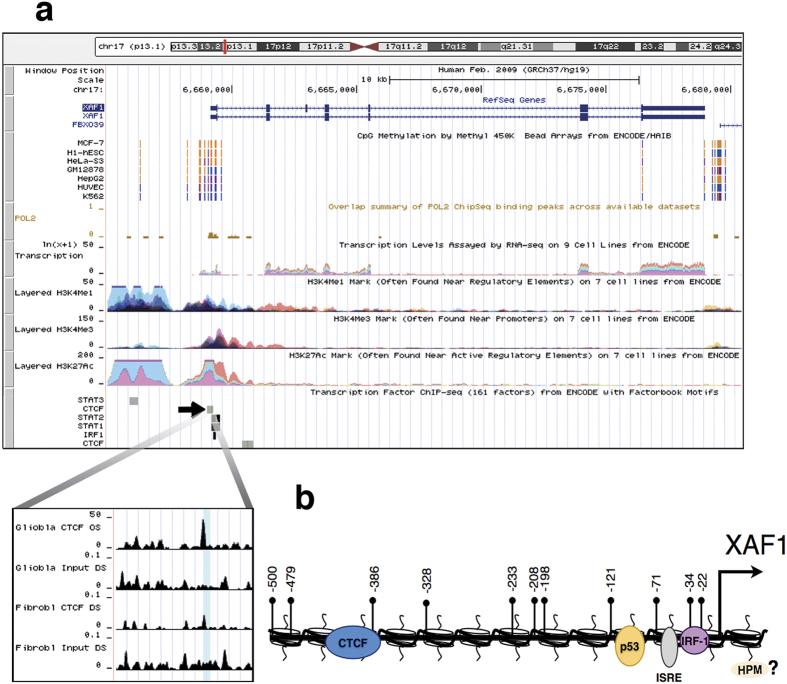
Features of the *XAF1* promoter. (**a**) The *XAF1* promoter visualized in the UCSC genome browser. The picture illustrates the CpG-methylation status from different types of cell lines. Additionally, the profiles of several histone posttranslational modifications such as H3K4Me1, H3K4Me3 and H3K27Ac are presented from different cell lines. Several transcription factor binding sites obtained from ChIP-Seq data are also shown. At a higher resolution, the CTCF binding site in the *XAF1* promoter in glioblastoma and fibroblast cells is shown. (**b**) Schematic representation of the *XAF1* promoter showing the CpG-dinucleotide positions from −22 to −500 bp relative to the transcription start site and the previously described binding sites for IRF-1, ISRE, p53 and the uncharacterized CTCF binding site. Histone posttranslational modification (HPM).

CTCF is known to regulate the expression of diverse tumour suppressor genes by directly binding to promoter sequences^28^. We searched for transcription binding sites in a window of −/+ 10 bp of the DNA sequence adjacent to each CpG that was demethylated as a consequence of epigenetic modifiers. Interestingly, we identify a putative CTCF binding site that overlapped the CpG dinucleotide located at −388 bp relative to the transcription start site (Fig. 2b). Supporting the relevance of this site, its presence was confirmed in an experimentally validated CTCF-binding site database^33^ (Fig. 3a,b). To experimentally validate this, ChIP assays were performed in MCF-7 cells after stimulation with TNF-α or IFN-α. As shown in Fig. 2c, in basal conditions, we could not find a detectable association of CTCF with the putative CTCF binding site in the *XAF1* promoter. This result could be explained by a methylation-sensitive CTCF binding mechanism. To directly test this, we exposed the cells to demethylating agents before stimulation with TNF-α or IFN-α. As expected, the association of CTCF with the *XAF1* promoter was detected only after DNA demethylation and stimulation with TNF-α or IFN-α (Fig. 2c). This observation correlated with an increased transcriptional activation when the cells were previously exposed to the epigenetic modifiers (Fig. 2c, third panel). Additionally, we validated this CTCF binding site using an additional cell line. As in MCF-7 cells, we observed a dramatic increase in the interaction of CTCF with the *XAF1* promoter when the cells were stimulated with either TNF-α or IFN-α after exposure to demethylating agents (Supplementary Fig. 1c). These results support a methylation-sensitive association of CTCF with the *XAF1* promoter.

### XAF1 expression is regulated by CTCF

**Figure 4:**
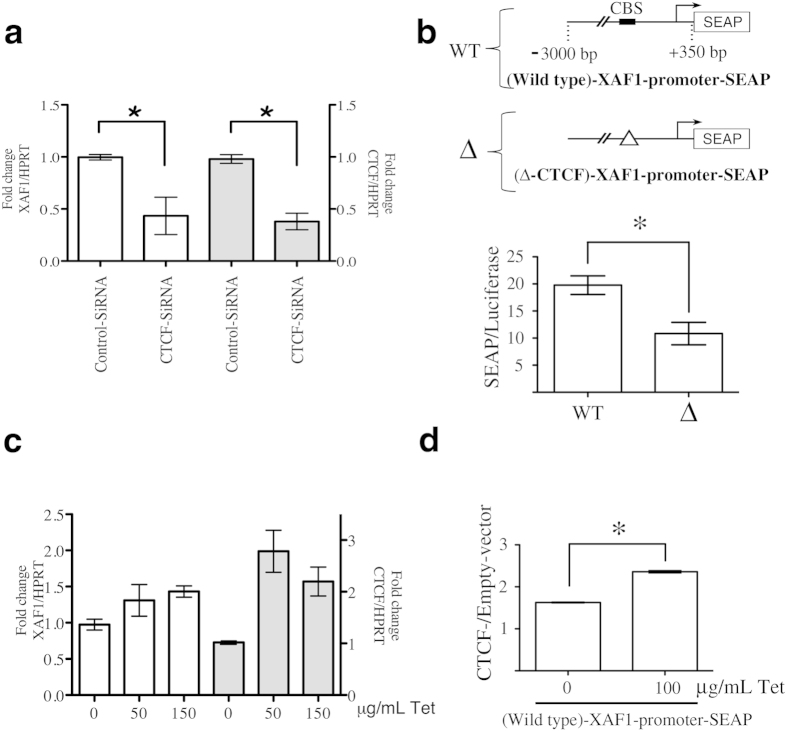
CTCF regulates transcriptional activation of the *XAF1* gene. (**a**) ACHN cells were pre-treated with 5-aza-2′-deoxycytidine (5 μM) and Trichostatin-A (0.2 μM) for 3 days. After that, the cells were transiently transfected with CTCF siRNAs or control scramble siRNAs. qPCR analyses were performed to measure the expression of both *XAF1* and *CTCF* mRNA. HPRT was used as loading control. The means from three independent experiments were plotted with +SEM, *P < 0.05. (**b**) MCF-7 cells were transitorily co-transfected with both Wild-type-XAF1-promoter-SEAP or Δ-CTCF-XAF1-promoter-SEAP constructs and pMetLuc, which was used for transfection normalization. Data are represented as the means + SEM from three independent experiments, *P < 0.05. (**c**) MCF-7 stable clones of CTCF/Tet-On were stimulated with tetracycline at the indicated concentrations. Using qPCR assays, *XAF1* and *CTCF* mRNA expression was normalized to *HPRT*, used as a loading control. The mean and range were plotted from two independent stable cell lines. (**d**) MCF-7-CTCF/Tet-On and MCF-7 Empty/Tet-On cell lines were transitory co-transfected with Wild-type-XAF1-promoter-SEAP and pMetLuc. After 48 h, tetracycline was added for 24 h. Data are represented as the means + SEM from three independent experiments, *P < 0.05. 5*-*Aza*-*2′-deoxycytidine (5-A-DC); Trichostatin-A (TSA); CTCF-binding site (CBS).

To further define the role of CTCF on *XAF1* mRNA expression, we used specific siRNAs to downregulate CTCF expression in a series of loss-of-function experiments. We verified the efficacy of these siRNAs at both mRNA and protein levels (Supplementary Fig. 1d). Because previous reports have shown that demethylating agents increase XAF1 induction by IFN in ACHN cells^32^, we used this cell line to analyse the effect of these siRNAs on XAF1 transcriptional responsiveness to TNF-α or IFN-α. As described above, demethylating conditions are necessary to uncover the CTCF-binding site (Fig. 2b,c). We clearly observed lower levels of *XAF1* mRNA in cells transfected with the siRNAs against CTCF than those transfected with control siRNAs (Fig. 4a). Additionally, we confirmed the regulatory effect of CTCF on the *XAF1* promoter using the secreted alkaline phosphatase (SEAP) reporter gene assays. In these assays, the enzymatic activity drove by the *XAF1* promoter region comprising −3000 bp to +350 bp relative to the transcription start site (Wild-type-XAF1-promoter-SEAP) was compared with the same region with a deletion of the core CTCF binding site (Δ-CTCF-XAF1-promoter-SEAP). The absence of the CTCF binding site in the XAF1 promoter inhibits its basal transcriptional activation (Fig. 4b). To further support these results, we also conducted gain-of-function experiments by analysing the effects of CTCF overexpression on *XAF1* mRNA expression. To achieve this, we engineered a Tet-on CTCF system in the MCF-7 cell line. In demethylating conditions, the overexpression of CTCF mediated by tetracycline addition induced transcriptional activation of XAF1 (Fig. 4c). Additionally, these cells were transfected with the Wild-type-XAF1-promoter-SEAP construct. After tetracycline addition, we observed a significant increase in the enzymatic activity of the reporter in cells overexpressing CTCF (Fig. 4d). On the other hand, we evaluated the role of CTCF over-expression in terms of transcriptional responsiveness of XAF1 in TNF-α- or IFN-α–treated cells. Although we detected an increase in XAF1 levels in cells over-expressing CTCF, the TNF-α or IFN-α-mediated transcriptional increase was not modified by CTCF overexpression (supplementary Fig. 2a). This points toward a shared signalling mechanism and supports the role of CTCF in the effects of these cytokines on XAF1 regulation, with additional factors needed for maximal responsiveness. Thus, both gain and loss of function approaches showed the participation of CTCF in XAF1 expression.

### CTCF protects the XAF1 gene from epigenetic silencing

**Figure 5:**
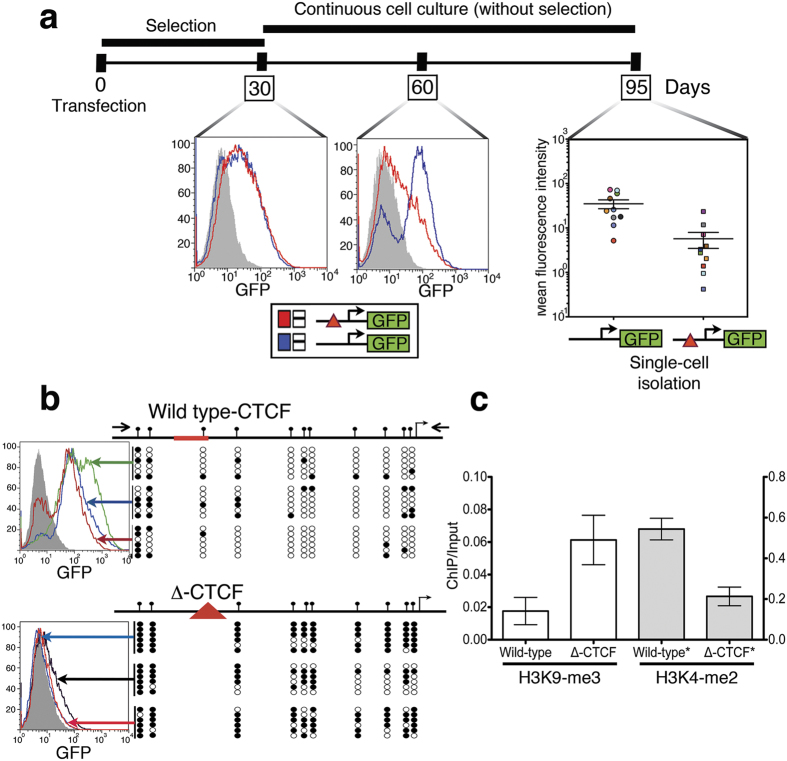
CTCF maintains an open-chromatin configuration in the *XAF1* promoter in transgene assays. (**a**) The timeline for stable transgenic cell line generation using peGFPN1-XAF1 or peGFPN1-Δ-CTCF-XAF1 plasmids. The detection of GFP expression for each cell line was performed using FACS. Single-cell clones were generated at day 60. (**b**) Right, bisulphite sequencing was performed from single-cell clones containing either peGFPN1-XAF1 or peGFPN1-Δ-CTCF-XAF1 constructs. The exogenous *XAF1* promoter was specifically amplified using a nested-PCR strategy in which the first amplification was performed using primers recognizing plasmid sequences. Methylated and unmethylated CpGs are depicted as filled and open circles, respectively. Left, histograms from each single cell clones are showed (**c**) ChIP assays were performed from stable single-cell clones using specific antibodies against H3K4-2me or H3K9-3me. Data are represented as the means ± SEM from three single-cell clones. Red triangle symbols the deletion of the CTCF binding site.

The insulating action of CTCF protects several genes from epigenetic silencing^28^,^34^. In particular, it has been described that the absence of CTCF in tumour suppressor gene promoters induces their epigenetic silencing, which supports the role of CTCF in cancer^35^,^36^,^37^. To test the possible epigenetic-mediated regulation of CTCF on the *XAF1* gene, we compared the *XAF1* promoter activity in a genomic integrated context by measuring a *GFP* reporter gene. For this, we compared the wild type *XAF1* promoter with the CTCF-deletion (Δ-CTCF-XAF1) construct. Supporting the insulating role of CTCF on the *XAF1* gene, cells with the Δ-CTCF-XAF1 promoter showed lower GFP levels than those with the wild-type *XAF1* promoter after 60 days of continuous culture (Fig. 5a). To further support this finding, single-cell clones for each transfection were isolated and propagated for an additional 35 days. As expected by the previous result, silencing of GFP expression levels was mainly observed in single-cell clones with the integrated Δ-CTCF-XAF1-promoter (Fig. 5a), pointing toward an epigenetic-protective effect of the CTCF binding site. A possible alternative explanation for the difference in GFP expression levels between transfections could be attributed to a distinct number of integration events. To exclude this possibility, the transgene copy number was measured by real-time PCR as previously reported^38^. The difference between GFP expression levels driven by the Δ-CTCF-XAF1-promoter and wild-type-XAF1-promoter was independent of the transgene copy number (Supplementary Fig. 2b). To gain insight into the epigenetic mechanism involved in GFP silencing of the Δ-CTCF-XAF1-promoter, we hypothesized that loss of the CTCF-binding site could promote 1) accelerated CpG methylation or 2) acquisition of a repressive chromatin configuration based on HPMs (or both). To test the first hypothesis, sequencing of the sodium bisulphite-modified genomic DNA from three single-cell clones for each transfection was performed. To discriminate endogenous *XAF1* promoter amplification, a nested-PCR strategy was performed in which the first set of primers annealed to plasmid sequences surrounding the exogenous *XAF1* promoter (Fig. 5b and Supplementary Fig. 2c). We observed that the Δ-CTCF-XAF1-promoter is more susceptible to dinucleotide-CpG methylation than the wild-type-XAF1-promoter in a genomic-integrated context (Fig. 5b). To test our second hypothesis, ChIP assays were performed using specific antibodies directed to H3K4-2me or H3K9-3me posttranslational modifications in single-cell clones from each stable transfection. To interrogate the relative enrichment of repressive or active HPMs in the transgene, we designed a pair of primers that anneal to the plasmid sequence immediately after the exogenous *XAF1* promoter (Fig. 5c and Supplementary Fig. 2c). Lower levels of the H3K4-2me posttranslational modification, a marker for transcription activity, were observed in cell single clones from the Δ-CTCF XAF1-promoter (Fig. 5c). As expected, the Δ-CTCF XAF1 promoter was enriched with the repressive H3K9-3me modification (Fig. 5c). Overall, these findings support the notion that CTCF regulates DNA methylation in the XAF1 promoter; thus, loss of CTCF in its cognate-binding site induces DNA-methylation and polarization from active to repressive HPM, which in turn induces transcriptional repression.

### XAF1 expression is modulated by CTCF in apoptotic conditions

**Figure 6:**
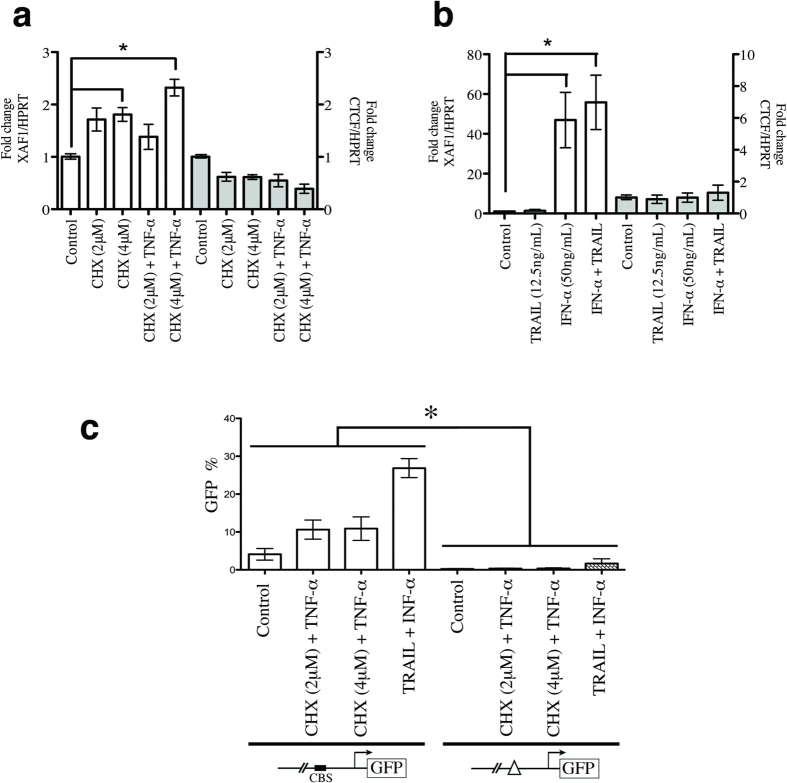
The CTCF binding site in the *XAF1* promoter mediates XAF1 responsiveness to activators in apoptotic conditions. (**a**) MCF-7 cells were treated with 5-Aza-2′-deoxycytidine (5 μM) and Trichostatin-A (0.2 μM) for 3 days before stimulation with TNF-α in the presence of cycloheximide (TNF-α + CHX) (left panel). qPCR analyses of *XAF1* and *CTCF* mRNA expression were performed. *HPRT* mRNA was used as loading control. Results are presented as fold change. Data are represented as the means ± SEM from three independent experiments, *P < 0.05. (**b**) MCF-7 cells were pre-treated as shown in (A) before the addition of IFN-α in the presence of TRAIL (IFN-α + TRAIL) (right panel). The expression of *XAF1* and *CTCF* and *HPRT* was determined by qPCR. *HPRT* was used as loading control. (**c**) Stable single-cell clones containing either peGFPN1-XAF1 or peGFPN1-Δ-CTCF-XAF1 constructs were stimulated with either TNF-α + CHX or IFN-α + TRAIL. After, GFP protein levels were measured using FACS. Data are represented as the mean SD of four single-cell clones from each transfection, *P < 0.05.

It has been well described that XAF1 expression reactivation has a crucial role in apoptosis induced by TNF-α/cycloheximide (CHX) or IFN-α/TNF-related apoptosis-inducing ligand (TRAIL)^6^,^22^. To test if CTCF could regulate XAF1 expression in apoptotic conditions, MCF-7 cells were exposed to either TNF-α/CHX or IFN-α/TRAIL. Cytotoxicity induced by the co-treatment of either TNF-α/CHX or IFN-α/TRAIL was analysed by cell viability assays (Supplementary Fig. 2d). As expected, we observed the transcriptional activation of XAF1 after exposure to both regimens (Fig. 6a,b). To assess the biological relevance of CTCF-mediated XAF1 transcription, single-cell clones with the wild type- or Δ-CTCF-XAF1 promoter were exposed to inducers of apoptosis. After that, FACS was used to measure GFP-reporter gene activity. Whereas the wild-type promoter activity correlated with the XAF1 transcriptional activation, the Δ-CTCF-XAF1 promoter did not present any transcriptional activity (Fig. 6c). Several reports have shown that XAF1 is an IFN-stimulated gene in cancer cells^30^,^39^,^40^. Because its promoter is commonly hypermethylated in these cells, transcriptional activation of the XAF1 gene could be dependent on IFN-α-mediated demethylation and could thus rely on CTCF^20^. Supporting this hypothesis, we found that single-cell clones with the Δ-CTCF-XAF1 promoter were unable to respond to IFN-α, indicating that CTCF could be relevant in the IFN-α-mediated induction of XAF1 (Fig. 6c).

## Discussion

In cancer, it has been described that the *XAF1* gene is transcriptionally silenced by CpG-dinucleotide hypermethylation in its promoter^9^,^17^,^41^. Exposure to demethylating agents induces XAF1 transcriptional activation, thereby compromising cell viability by promoting apoptosis^4^,^21^, mitotic catastrophe^4^,^8^ or cell cycle inhibition^8^. Thus, CpG methylation in the *XAF1* promoter represents the main epigenetic mechanism involved in XAF1 silencing and, consequently, in resistance against apoptosis. However, the deregulation of epigenetic mechanism is implicated in a variety of diseases^42^, including cancer^43^,^44^,^45^. CTCF is a multi-task protein involved in chromatin regulation, with profound consequences in gene expression^23^,^24^. In a panel of breast cancer cell lines, heightened CTCF expression was associated with apoptosis resistance^46^. The protective action of CTCF is explainable, in part, by negative regulation of the *Bax* gene, which increases the apoptotic threshold^47^. It has also been clearly demonstrated that CTCF regulates the chromatin configuration of many tumour suppressor genes, affecting their transcription rates^28^. Here, we describe that CTCF interacts with the *XAF1* promoter, thereby regulating its chromatin configuration and, consequently, its transcriptional responsiveness to well-known activators. We were able to demonstrate two biological scenarios. First, CTCF maintains an open-chromatin configuration in the *XAF1* promoter, as assessed by the presence of both active HPMs (Fig. 5c) and de-methylated CpG dinucleotides (Fig. 5b), allowing high transcriptional responsiveness to activators (Figs 1 and 6c). Second, the loss of CTCF regulation in the *XAF1* promoter, explained by the fact that CTCF interaction with its cognate binding site in the *XAF1* promoter is methylation sensitive (Fig. 2b,c), induces polarization from active to repressive HPMs (Fig. 5c) and accelerates CpG-dinucleotide methylation (Fig. 5b). This closed chromatin state represses transcriptional activation (Fig. 6c) and possibly contributes to apoptotic resistance. Thus, CTCF is a determinant that confers a permissive chromatin configuration to the *XAF1* gene, which is critical for apoptotic program culmination.

In X-chromosomal inactivation, certain genes escape from the epigenetic silencing mechanism. CTCF mediates this escape by inhibiting the propagation both of methylation and of repressive HPMs from surrounding silent regions^34^. In this scenario, CTCF mediates the shift between an open and closed chromatin configuration by functioning as a scaffold protein to attract different enzymatic complexes involved in HPMs^35^,^48^,^49^. Concordantly, epigenetic silencing of CTCF-regulated genes is observed when CTCF is unable to interact with its DNA-binding site by a methylation-sensitive interaction that favours the presence of repressive, HPMs^27^,^35^,^50^. Importantly, reports showing the biological weight of different HPMs in the *XAF1* promoter are missing. In the present report, assessing long-term transgenic behaviour allowed us to uncover the actions of CTCF on the chromatin configuration of the *XAF1* promoter (Fig. 5). We observed for the first time that the absence of local CTCF in the *XAF1* promoter induces the transition from active to repressive HPMs. We envision that the loss of CTCF affinity to its cognate binding-site in the XAF1 promoter could be the first driving event for the transcriptional repression of the XAF1 gene. Additional experiments are needed to support this, but it has been reported that CTCF posttranslational modifications impair its ability to interact with DNA targets^51^,^52^,^53^,^54^. Thus, lack of CTCF in its cognate site allows its methylation, impeding re-association of CTCF to it even with new posttranslational modifications arise. Consequently, this induces accelerated CpG-dinucleotide methylation and polarization from active to repressive HPM and a consequent XAF1 transcriptional silencing.

**Figure 7:**
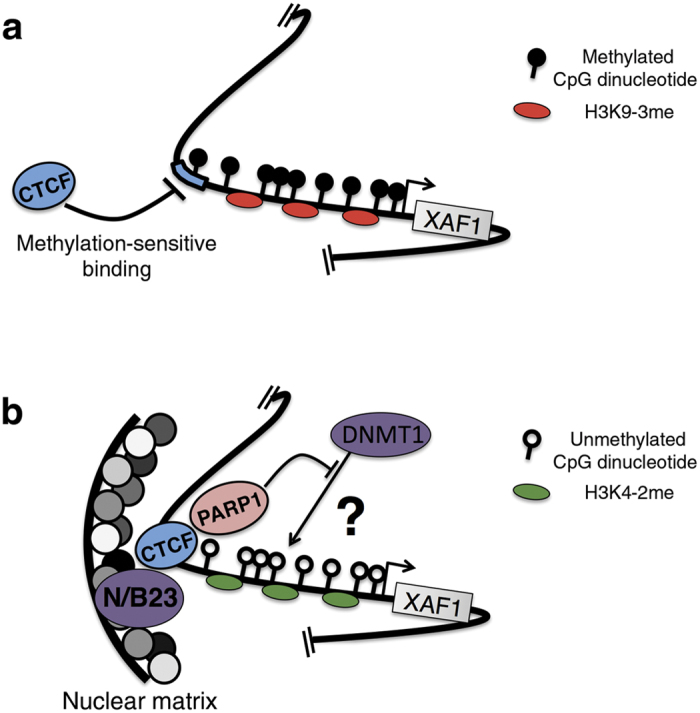
Epigenetic regulation by CTCF of the *XAF1* promoter. (**a**) Methylation of the CTCF binding site in the *XAF1* promoter, as occurs in cancer, inhibits its recognition by CTCF. This enriches repressive histone posttranslational modifications, contributing to XAF1 transcriptional silencing. (**b**) In demethylating conditions, CTCF is able to interact with its cognate DNA-binding site, inhibiting both CpG-dinucleotide methylation and repressive histone posttranslational modifications such as H3K9-2me. After treatment with transcriptional activators such as TNF-α or IFN-α, the association between CTCF and nucleophosmin/B23 is induced, allowing *XAF1* promoter anchorage to the nuclear matrix and inducing transcriptional activation. Additionally, several subunits of CTCF could bridge additional genomic regions from interchromosomal or intrachromosomal locations to the same genomic anchor to which the *XAF1* promoter is attached. The inhibition of CpG-dinucleotide methylation could be mediated by the inhibitory action of PARP-1 on DNMT1.

It has been described that the nuclear matrix plays an important role in the regulation of gene transcription. Chromatin is anchored by short stretches of DNA sequences called matrix attachment regions (MARs). MARs range in size from 100 to 2000 bp and are rich in AT dinucleotide pairs and repetitive sequences. Both chromatin loop formation and the transcriptional activation of genes surrounded by MARs are dependent on nuclear matrix anchorage^55^. This is explainable by the fact that transcriptional factors are present in the nuclear matrix^56^. CTCF associates with the nuclear matrix^57^,^58^ and mediates the anchoring of DNA sequences to it, as observed in the 5′-HS4 chicken β-globin insulator^58^. In addition, the association of CTCF with the nuclear matrix depends on nucleophosmin/B23^59^. However, it has been observed that IFN-γ induces transcriptional activation of major histocompatibility complex genes, which coincides with the reorganization of chromatin loops^60^. Interestingly, DNA anchorage to the nuclear matrix after IFN-γ exposure was associated with CTCF binding sites^60^. Therefore, CTCF reconfigures genomic regions by forming loops that affect the transcriptional gene landscape. In the present paper, we demonstrate that in cancer cells, CTCF is unable to associate with its cognate DNA-binding site in the *XAF1* promoter if it is methylated (Fig. 7a), thus effectively rendering it unresponsive to well-known activators (Fig. 7a). However, after demethylating the cognate site, CTCF is now able to associate with the *XAF1* promoter to enhance transcriptional activation (Fig. 2). One intriguing possibility is that CTCF could be able to attract DNA to the nuclear matrix, mediating faster chromatin loop formation in the nuclear matrix after exposure to exogenous stimuli. Although not tested, we envision that CTCF could be able to attract the *XAF1* promoter to the nuclear matrix by its association with nucleophosmin/B23, thereby inducing both chromatin loop formation and transcriptional activation of the *XAF1* gene (Fig. 7b). In cancer, this putative mechanism would not occur due to the absence of CTCF in its DNA-binding site via a methylation-sensitive mechanism (Fig. 7a).

Finally, we demonstrate for the first time that CTCF is critical to maintaining key CpG-dinucleotides demethylated in the *XAF1* promoter (Fig. 5b). This could be explainable by previous reports showing that CTCF associates with and activates PARP-1, which negatively regulates DNMT1, thus maintaining the CpG dinucleotides within the CTCF-binding sites free from methylation^61^,^62^. Additionally, a pool of PARP is located in the nuclear matrix and is implicated in chromatin loop formation. Although not tested, an interesting hypothesis would be the possibility that PARP-1 is a mediator of the effects of CTCF (Fig. 7b). Further experiments are required to test this.

In conclusion, we demonstrate a novel functional CTCF binding site in the *XAF1* promoter. The association of CTCF with its binding site induces an open chromatin configuration by enriching active HPMs and maintaining CpG-dinucleotides free from methylation. In cancer, methylation negatively affects the interaction between CTCF and the *XAF1* promoter, disabling the protective epigenetic actions of CTCF against the closed-chromatin configuration. Our finding are consistent with CTCF acting as key regulatory element in the well-accepted observation that CpG-dinucleotide methylation on the *XAF1* promoter inhibits its transcriptional activation. The absence of CTCF regulation of the *XAF1* gene may constitute a selective advantage during clonal evolution by means of increasing the apoptotic threshold.

## Methods

### Cell culture and reagents

MCF-7 (HTB22) cells were acquired from the American Type Culture Collection (ATCC) and maintained in Dulbecco’s modified Eagle’s medium (DMEM) supplemented with 10% fetal bovine serum (FBS). ACHN (CRL-1611) cells were acquired from the ATCC, and maintained in Eagle’s Minimum Essential Medium (EMEM) supplemented with 10% FBS. The cells were grown in a humidified incubator that was maintained at 37 °C with 5% CO_2_. Demethylating conditions were established by treating the cell lines for 3 days with 0.2 μM Trichostatin-A (TSA) and 5 μM 5-aza-2′-deoxycytidine (5-A-DC) (SIGMA). Daily, the medium was replaced with fresh medium containing 5-A-DC and TSA. The transfection of constructs was performed using Lipofectamine 2000 (Invitrogen). TNF-α and IFN-α were purchased from R&D and PROSPEC, respectively.

### Constructs

Genomic DNA isolated from peripheral human blood was used as a template. Primers used in this work are listed in Supplementary Table S1. Specific primers were designed to amplify by PCR the region from −1200 to +350 bp relative to the transcription start site (TSS) from the *XAF1* gene (XAF1.2). The PCR product was purified and cloned into pTZ57r/t (Thermo). Then, XAF1.2 was subcloned into the peGFP-N1 (Clontech) expression vector to produce peGFP-N1-XAF1-promoter. Deletion of the CTCF-binding site from the peGFP-N1-XAF1-promoter plasmid was performed using the QuickChange Lightning Site-Directed Mutagenesis kit (Agilent Technologies). Following the manufacturer’s protocol, we generated peGFP-N1-Δ-CTCF-XAF1. Specific primers were designed to amplify by PCR the genomic region from −3000 to +350 bp relative to the transcription start site from the *XAF1* gene (XAF1-promoter). The PCR product was cloned using GeneJET PCR cloning kit (Fermentas) and was then subcloned into pSEAP2-Basic (Clontech), a secreted alkaline phosphatase (SEAP) gene reporter expression vector, to produce the wild-type-XAF1-promoter-SEAP construct. Using a QuickChange Lightning Site-Directed Mutagenesis kit (Agilent Technologies), we generated the Δ-CTCF-XAF1-promoter-SEAP construct, which lacked the CTCF binding site. To generate an inducible system for CTCF overexpression, CTCF was amplified from cDNA using Pfu polymerase (Stratagene) and cloned into pQCXIP (Clontech). It was then subcloned into pTRE-Tight-Bi-AcGFP1 (Clontech) to produce pTRE-Tight-Bi-AcGFP1-CTCF. All plasmids were confirmed by capillary sequencing.

### Transient and stable transfection of MCF-7 cells

MCF-7 cells were seeded in 12-well plates one day before transfection. The cells were co-transfected with 0.625 μg of either wild-type-XAF1-promoter-SEAP or Δ-CTCF-XAF1-promoter-SEAP plasmids and 0.625 pg of pMetLuc (Clontech), which is a plasmid encoding secreted Metridia luciferase used for transfection normalization. After 24 h, the transfection medium was changed out for fresh medium. After 48 h, the medium was collected to measure both SEAP and Luciferase activities using the Great EscAPe SEAP chemiluminescence kit (Clontech) and Ready-To-Glow-Secreted Luciferase Reporter Assay (Clontech), respectively.

For inducible CTCF overexpression, MCF-7 cells were transfected with 2 μg pTet-On plasmid (Clontech), which encodes the rTet repressor protein. The cells were selected in G418 (1000 μg/mL) for 4 weeks. The pool of the resulting colonies was then expanded under G418 selection and cotransfected with 2 μg pTRE-Tight-Bi-AcGFP1-CTCF with 1 μg pQPCXIP empty plasmid. Stable cell clones were selected with puromycin after two weeks of selection.

MCF-7 cells were seeded in 6-well plates. After 1 day, the cells were transfected with 2 μg of either peGFP-N1-XAF1-promoter or peGFP-N1-Δ-CTCF-XAF1-promoter plasmids. After 48 h, the cells were selected with G418 (1000 μg/mL) for 4 weeks. Then, G418-resistan cells were analysed by fluorescence activate cell sorting (FACS). The resistant cells were further cultured for 30 days in the absence of G418 and were analysed by FACS. Then, single cell clones were isolated. The single cell clones were continuously cultured further in the absence of G418 for 35 days, and reporter gene expression was evaluated by FACS.

### Transient transfection of small interfering RNAs (siRNAs) against CTCF

ACHN and MCF-7 cells were seeded in 6-well plates. After one day, the cells were treated in demethylating conditions, as indicated above. The cells were then transfected with 0.1 μM human CTCF small interfering RNAs (siRNAs; Qiagen) using Lipofectamine® 2000 (Invitrogen). After 24 h, the transfection medium was replaced with fresh medium containing demethylating agents. RNA isolation was performed 48 h post-transfection using TRIzol reagent (Invitrogen). RNA was converted to cDNA using random primers and SuperScript® VILO (Invitrogen).

### Bisulphite DNA sequencing analysis

DNA was extracted from either MCF-7 cells or MCF-7 stable cell lines using the FlexiGene DNA Kit (Qiagen). DNA (1.5 μg) was bisulphite converted using the Zymo EZ DNA Methylation Kit (Zymo Research) according to the manufacturer’s protocol. The bisulphite-converted DNA was subjected to PCR amplification using specific primers to the *XAF1* promoter (XAF-EnBis/XAF-EnBia). A nested-PCR amplification strategy was used to amplify DNA converted from stable cell lines using specific primers against the plasmid sequence (1.2GFPBis/1.2GFPBia) in the first PCR reaction to avoid amplification of the *XAF1* endogenous promoter. The product from this PCR was used in a second round of PCR amplification using specific primers against the *XAF1* promoter, as described above. PCR products were gel purified and cloned using the GeneJET PCR cloning kit (Fermentas), and positive clones were sent for sequencing.

### Chromatin immunoprecipitation

Cells (3 × 10^6^) were fixed with 1% formaldehyde and neutralized by adding 0.125 M glycine. The cells were then lysed in cell lysis buffer (10 mM EDTA, 50 mM TRIS-HCl pH 8, 1% SDS, protease inhibitor cocktail). The cell lysate was sonicated to obtain soluble chromatin with a mean length of 400 bp. Chromatin immunoprecipitation (ChIP) was performed using a specific antibody raised against CTCF (C02-2899; Cell Signaling Technology) or CTCF (07-729; Millipore). Specific antibodies against H3K9me3 (ab8898 Abcam) and H3K4me2 (7766 Abcam ab) were used to perform ChIP assays on soluble chromatin from single cell clones. The DNA recovered after ChIP was subjected to PCR amplification using the following primers: XAF-CTCF-s/XAF-CTCF-as was used for the putative CTCF binding site of the XAF1 promoter; two set of primers, IGF2-CTCF-s/IGF2-CTCF-as^63^ and MYC-CTCF-s/MYC-CTCF-as^64^, were used as positive controls for the CTCF-DNA interaction; and a negative control, NEG-CTCF-s/NEG-CTCF-as, was also included.

### Immunoblotting

Protein fractions were subjected to either 15% or 18% SDS-PAGE and transferred to Immobilon P membranes (Millipore). Next, the membranes were incubated with the indicated antibodies overnight, and the blots were visualized using the Immobilon Western kit (Millipore) with a peroxidase-labelled secondary antibody, according to the manufacturer’s protocols.

### Protein immunoprecipitation assay

The cells were washed with PBS, scraped and centrifuged at 2,000 rpm for 3 min. The cells were lysed using 1 mL of TNTE-5 buffer (50 mM Tris pH 7.4, 150 mM NaCl, 0.5% Triton, 1 mM EDTA and 1X protease inhibitor cocktail) and incubated at 4 °C for 15 min. The lysates were centrifuged at 14000 *g* for 10 min. The supernatants were incubated with 10 μL of recombinant protein G agarose beads (Life Technologies) for 1 h. After incubation, the lysates were centrifuged at 14000 *g* for 30 sec. The supernatants were incubated overnight at 4 °C with 3 μL of primary antibody with constant agitation. Next, 20 μL of recombinant protein G agarose beads was added to each lysate, and the lysates were then incubated with constant agitation for 1 h on ice. The lysates were next centrifuged at 14000 *g* for 10 sec. The resulting pellets were washed twice with TNTE-1 buffer (50 mM Tris at pH 7.4, 150 mM NaCl, 0.1% Triton, 1 mM EDTA and 1X protease inhibitor cocktail), followed by boiling in Laemmli sample solution (100 mM Tris pH 6.8, 20% Glycerol, 2% SDS, 0.05% bromophenol blue and 100 mM DTT) for further analysis.

### Cell viability assay

Cell viability was measured colorimetrically using the MTS-PMS assay (CellTiter 96® Aqueous Non-Radioactive Cell Proliferation Assay; Promega) according to the manufacturer’s protocol. Briefly, the cells were seeded in 96-well plates, and 24 h after treatment, the reagents from the kit were added to the culture medium. After 2 h of incubation, the absorbance was measured at a wavelength of 490 nm using a microplate reader.

### RT-qPCR

Total RNA was extracted using TRIzol reagent (Invitrogen) according to the manufacturer’s instructions. Briefly, 2 μg of total RNA was used for cDNA synthesis with random hexamers. Quantitative PCR was carried out using an ABI PRISM 7900 Sequence Detection System (Applied Biosystems) using IDT Prime Time qPCR Primers and ZEN Double-Quenched Probe for detecting XAF1, CTCF, and HPRT genes. The TaqMan Universal PCR Master Mix (Applied Biosystems) was used. The geometric mean of housekeeping gene HPRT was used as an internal control to normalize the variability in expression levels. Results were analyzed by the comparative 2 ^−ΔΔCT^ method to calculate fold changes in gene expression^65^. The plotted results include the mean + s.e.m. from at least three independent biological experiments.

### Statistical analysis

GraphPad Prism version 5.0 for Mac Os X (La Jolla, CA) was used to perform statistical analyses. One-way analysis of variance was performed, and the Bonferroni post-test was used at 95% confidence intervals to determine significant differences.

## Additional Information

**How to cite this article**: Victoria-Acosta, G. *et al.* Epigenetic silencing of the XAF1 gene is mediated by the loss of CTCF binding. *Sci. Rep.* **5**, 14838; doi: 10.1038/srep14838 (2015).

## Supplementary Material

Supplementary Figures

## References

[b1] Liston P. *et al.* Identification of XAF1 as an antagonist of XIAP anti-Caspase activity. Nat Cell Biol 3, 128–133 (2001). 11175744 10.1038/35055027

[b2] Kim K. S., Heo J. I., Choi K. J. & Bae S. Enhancement of cellular radiation sensitivity through degradation of Chk1 by the XIAP-XAF1 complex. Cancer Biol Ther 15, 1622–1634 (2014). 25535897 10.4161/15384047.2014.962305 PMC4623052

[b3] Tse M. K. *et al.* Domain organization of XAF1 and the identification and characterization of XIAP(RING) -binding domain of XAF1. Protein Sci 21, 1418–1428 (2012). 22811387 10.1002/pro.2126 PMC3526985

[b4] Arora V. *et al.* Degradation of survivin by the X-linked inhibitor of apoptosis (XIAP)-XAF1 complex. J Biol Chem 282, 26202–26209 (2007). 17613533 10.1074/jbc.M700776200

[b5] Fong W. G. *et al.* Expression and genetic analysis of XIAP-associated factor 1 (XAF1) in cancer cell lines. Genomics 70, 113–122 (2000). 11087668 10.1006/geno.2000.6364

[b6] Xia Y., Novak R., Lewis J., Duckett C. S. & Phillips A. C. Xaf1 can cooperate with TNFalpha in the induction of apoptosis, independently of interaction with XIAP. Mol Cell Biochem 286, 67–76 (2006). 16432762 10.1007/s11010-005-9094-2

[b7] Zou B. *et al.* XIAP-associated factor 1 (XAF1), a novel target of p53, enhances p53-mediated apoptosis via post-translational modification. Mol Carcinog 51, 422–432 (2012). 21678496 10.1002/mc.20807

[b8] Wang J. *et al.* Identification of XAF1 as a novel cell cycle regulator through modulating G(2)/M checkpoint and interaction with checkpoint kinase 1 in gastrointestinal cancer. Carcinogenesis 30, 1507–1516 (2009). 19628579 10.1093/carcin/bgp155

[b9] Zou B. *et al.* Correlation between the single-site CpG methylation and expression silencing of the XAF1 gene in human gastric and colon cancers. Gastroenterology 131, 1835–1843 (2006). 17087954 10.1053/j.gastro.2006.09.050

[b10] Wang Y. *et al.* Association of expression of XIAP-associated factor 1 (XAF1) with clinicopathologic factors, overall survival, microvessel density and cisplatin-resistance in ovarian cancer. Regul Pept 178, 36–42 (2012). 22759793 10.1016/j.regpep.2012.06.005

[b11] Huang J. *et al.* XAF1 as a prognostic biomarker and therapeutic target in pancreatic cancer. Cancer Sci 101, 559–567 (2010). 19922503 10.1111/j.1349-7006.2009.01396.x PMC11158990

[b12] Chen X. Y., He Q. Y. & Guo M. Z. XAF1 is frequently methylated in human esophageal cancer. World J Gastroenterol 18, 2844–2849 (2012). 22719195 10.3748/wjg.v18.i22.2844 PMC3374990

[b13] Sakemi R. *et al.* X-linked inhibitor of apoptosis (XIAP) and XIAP-associated factor-1 expressions and their relationship to apoptosis in human hepatocellular carcinoma and non-cancerous liver tissues. Oncol Rep 18, 65–70 (2007). 17549347

[b14] Ng K. C., Campos E. I., Martinka M. & Li G. XAF1 expression is significantly reduced in human melanoma. J Invest Dermatol 123, 1127–1134 (2004). 15610524 10.1111/j.0022-202X.2004.23467.x

[b15] Fang X. *et al.* Switch to full-length of XAF1 mRNA expression in prostate cancer cells by the DNA methylation inhibitor. Int J Cancer 118, 2485–2489 (2006). 16353137 10.1002/ijc.21636

[b16] Kempkensteffen C. *et al.* Gene expression and promoter methylation of the XIAP-associated Factor 1 in renal cell carcinomas: correlations with pathology and outcome. Cancer Lett 254, 227–235 (2007). 17449173 10.1016/j.canlet.2007.03.006

[b17] Lee M. G. *et al.* Promoter CpG hypermethylation and downregulation of XAF1 expression in human urogenital malignancies: implication for attenuated p53 response to apoptotic stresses. Oncogene 25, 5807–5822 (2006). 16909101 10.1038/sj.onc.1209867

[b18] Chung S. K. *et al.* Frequent alteration of XAF1 in human colorectal cancers: implication for tumor cell resistance to apoptotic stresses. Gastroenterology 132, 2459–2477 (2007). 17570219 10.1053/j.gastro.2007.04.024

[b19] Lou Y. F. *et al.* Combination of gefitinib and DNA methylation inhibitor decitabine exerts synergistic anti-cancer activity in colon cancer cells. PLoS One 9, e97719 (2014). 24874286 10.1371/journal.pone.0097719 PMC4038521

[b20] Micali O. C. *et al.* Silencing of the XAF1 gene by promoter hypermethylation in cancer cells and reactivation to TRAIL-sensitization by IFN-beta. BMC Cancer 7, 52 (2007). 17376236 10.1186/1471-2407-7-52 PMC1845166

[b21] Zhu L. M. *et al.* Tumor suppressor XAF1 induces apoptosis, inhibits angiogenesis and inhibits tumor growth in hepatocellular carcinoma. Oncotarget 5, 5403–5415 (2014). 24980821 10.18632/oncotarget.2114 PMC4170645

[b22] Tu S. P. *et al.* Tumor suppressor XIAP-Associated factor 1 (XAF1) cooperates with tumor necrosis factor-related apoptosis-inducing ligand to suppress colon cancer growth and trigger tumor regression. Cancer 116, 1252–1263 (2010). 20082449 10.1002/cncr.24814

[b23] Phillips J. E. & Corces V. G. CTCF: master weaver of the genome. Cell 137, 1194–1211 (2009). 19563753 10.1016/j.cell.2009.06.001 PMC3040116

[b24] Ong C. T. & Corces V. G. CTCF: an architectural protein bridging genome topology and function. Nat Rev Genet 15, 234–246 (2014). 24614316 10.1038/nrg3663 PMC4610363

[b25] Filippova G. N. Genetics and epigenetics of the multifunctional protein CTCF. Curr Top Dev Biol 80, 337–360 (2008). 17950379 10.1016/S0070-2153(07)80009-3

[b26] Schoenherr C. J., Levorse J. M. & Tilghman S. M. CTCF maintains differential methylation at the Igf2/H19 locus. Nat Genet 33, 66–69 (2003). 12461525 10.1038/ng1057

[b27] Szabo P. E., Tang S. H., Silva F. J., Tsark W. M. & Mann J. R. Role of CTCF binding sites in the Igf2/H19 imprinting control region. Mol Cell Biol 24, 4791–4800 (2004). 15143173 10.1128/MCB.24.11.4791-4800.2004 PMC416431

[b28] Recillas-Targa F., De La Rosa-Velazquez I. A. & Soto-Reyes E. Insulation of tumor suppressor genes by the nuclear factor CTCF. Biochem Cell Biol 89, 479–488 (2011). 21846316 10.1139/o11-031

[b29] Straszewski-Chavez S. L. *et al.* XAF1 mediates tumor necrosis factor-alpha-induced apoptosis and X-linked inhibitor of apoptosis cleavage by acting through the mitochondrial pathway. J Biol Chem 282, 13059–13072 (2007). 17329253 10.1074/jbc.M609038200

[b30] Wang J. *et al.* All-trans retinoic acid induces XAF1 expression through an interferon regulatory factor-1 element in colon cancer. Gastroenterology 130, 747–758 (2006). 16530516 10.1053/j.gastro.2005.12.017

[b31] Sun Y. *et al.* Regulation of XAF1 expression in human colon cancer cell by interferon beta: activation by the transcription regulator STAT1. Cancer Lett 260, 62–71 (2008). 18035482 10.1016/j.canlet.2007.10.014

[b32] Reu F. J. *et al.* Overcoming resistance to interferon-induced apoptosis of renal carcinoma and melanoma cells by DNA demethylation. J Clin Oncol 24, 3771–3779 (2006). 16801630 10.1200/JCO.2005.03.4074

[b33] Ziebarth J. D., Bhattacharya A. & Cui Y. CTCFBSDB 2.0: a database for CTCF-binding sites and genome organization. Nucleic Acids Res 41, D188–D194 (2013). 23193294 10.1093/nar/gks1165 PMC3531215

[b34] Filippova G. N. *et al.* Boundaries between chromosomal domains of X inactivation and escape bind CTCF and lack CpG methylation during early development. Dev Cell 8, 31–42 (2005). 15669143 10.1016/j.devcel.2004.10.018

[b35] Batlle-Lopez A. *et al.* Novel CTCF binding at a site in exon1A of BCL6 is associated with active histone marks and a transcriptionally active locus. Oncogene 34, 246–256 (2015). 24362533 10.1038/onc.2013.535

[b36] Witcher M. & Emerson B. M. Epigenetic silencing of the p16(INK4a) tumor suppressor is associated with loss of CTCF binding and a chromatin boundary. Mol Cell 34, 271–284 (2009). 19450526 10.1016/j.molcel.2009.04.001 PMC2723750

[b37] Peng Z. *et al.* Epigenetic repression of RARRES1 is mediated by methylation of a proximal promoter and a loss of CTCF binding. PLoS One 7, e36891 (2012). 22615834 10.1371/journal.pone.0036891 PMC3355180

[b38] Joshi M., Keith Pittman H., Haisch C. & Verbanac K. Real-time PCR to determine transgene copy number and to quantitate the biolocalization of adoptively transferred cells from EGFP-transgenic mice. Biotechniques 45, 247–258 (2008). 18778249 10.2144/000112913

[b39] Leaman D. W. *et al.* Identification of X-linked inhibitor of apoptosis-associated factor-1 as an interferon-stimulated gene that augments TRAIL Apo2L-induced apoptosis. J Biol Chem 277, 28504–28511 (2002). 12029096 10.1074/jbc.M204851200

[b40] Leaman D. W. *et al.* Novel growth and death related interferon-stimulated genes (ISGs) in melanoma: greater potency of IFN-beta compared with IFN-alpha2. J Interferon Cytokine Res 23, 745–756 (2003). 14769151 10.1089/107999003772084860

[b41] Plenchette S., Cheung H. H., Fong W. G., LaCasse E. C. & Korneluk R. G. The role of XAF1 in cancer. Curr Opin Investig Drugs 8, 469–476 (2007). 17621877

[b42] Egger G., Liang G., Aparicio A. & Jones P. A. Epigenetics in human disease and prospects for epigenetic therapy. Nature 429, 457–463 (2004). 15164071 10.1038/nature02625

[b43] Feinberg A. P., Ohlsson R. & Henikoff S. The epigenetic progenitor origin of human cancer. Nat Rev Genet 7, 21–33 (2006). 16369569 10.1038/nrg1748

[b44] Esteller M. Epigenetics in cancer. N Engl J Med 358, 1148–1159 (2008). 18337604 10.1056/NEJMra072067

[b45] Baylin S. B. & Herman J. G. DNA hypermethylation in tumorigenesis: epigenetics joins genetics. Trends Genet 16, 168–174 (2000). 10729832 10.1016/s0168-9525(99)01971-x

[b46] Docquier F. *et al.* Heightened expression of CTCF in breast cancer cells is associated with resistance to apoptosis. Cancer Res 65, 5112–5122 (2005). 15958555 10.1158/0008-5472.CAN-03-3498

[b47] Mendez-Catala C. F. *et al.* A novel mechanism for CTCF in the epigenetic regulation of Bax in breast cancer cells. Neoplasia 15, 898–912 (2013). 23908591 10.1593/neo.121948 PMC3730042

[b48] Li T. *et al.* CTCF regulates allelic expression of Igf2 by orchestrating a promoter-polycomb repressive complex 2 intrachromosomal loop. Mol Cell Biol 28, 6473–6482 (2008). 18662993 10.1128/MCB.00204-08 PMC2577414

[b49] Soto-Reyes E. & Recillas-Targa F. Epigenetic regulation of the human p53 gene promoter by the CTCF transcription factor in transformed cell lines. Oncogene 29, 2217–2227 (2010). 20101205 10.1038/onc.2009.509

[b50] Eldholm V., Haugen A. & Zienolddiny S. CTCF mediates the TERT enhancer-promoter interactions in lung cancer cells: identification of a novel enhancer region involved in the regulation of TERT gene. Int J Cancer 134, 2305–2313 (2014). 24174344 10.1002/ijc.28570

[b51] Wang J., Wang Y. & Lu L. De-SUMOylation of CCCTC binding factor (CTCF) in hypoxic stress-induced human corneal epithelial cells. J Biol Chem 287, 12469–12479 (2012). 22354964 10.1074/jbc.M111.286641 PMC3320996

[b52] MacPherson M. J., Beatty L. G., Zhou W., Du M. & Sadowski P. D. The CTCF insulator protein is posttranslationally modified by SUMO. Mol Cell Biol 29, 714–725 (2009). 19029252 10.1128/MCB.00825-08 PMC2630690

[b53] Klenova E. M. *et al.* Functional phosphorylation sites in the C-terminal region of the multivalent multifunctional transcriptional factor CTCF. Mol Cell Biol 21, 2221–2234 (2001). 11238955 10.1128/MCB.21.6.2221-2234.2001 PMC86856

[b54] Venkatraman B. & Klenova E. Role of CTCF poly(ADP-Ribosyl)ation in the regulation of apoptosis in breast cancer cells. Indian J Med Paediatr Oncol 36, 49–54 (2015). 25810575 10.4103/0971-5851.151784 PMC4363851

[b55] Ottaviani D., Lever E., Takousis P. & Sheer D. Anchoring the genome. Genome Biol 9, 201 (2008). 18226181 10.1186/gb-2008-9-1-201 PMC2395230

[b56] Szentirmay M. N. & Sawadogo M. Spatial organization of RNA polymerase II transcription in the nucleus. Nucleic Acids Res 28, 2019–2025 (2000). 10773068 10.1093/nar/28.10.2019 PMC105382

[b57] Dunn K. L., Zhao H. & Davie J. R. The insulator binding protein CTCF associates with the nuclear matrix. Exp Cell Res 288, 218–223 (2003). 12878173 10.1016/s0014-4827(03)00185-x

[b58] Yusufzai T. M. & Felsenfeld G. The 5′-HS4 chicken beta-globin insulator is a CTCF-dependent nuclear matrix-associated element. Proc Natl Acad Sci USA 101, 8620–8624 (2004). 15169959 10.1073/pnas.0402938101 PMC423244

[b59] Yusufzai T. M., Tagami H., Nakatani Y. & Felsenfeld G. CTCF tethers an insulator to subnuclear sites, suggesting shared insulator mechanisms across species. Mol Cell 13, 291–298 (2004). 14759373 10.1016/s1097-2765(04)00029-2

[b60] Ottaviani D. *et al.* CTCF binds to sites in the major histocompatibility complex that are rapidly reconfigured in response to interferon-gamma. Nucleic Acids Res 40, 5262–5270 (2012). 22367884 10.1093/nar/gks158 PMC3384298

[b61] Guastafierro T. *et al.* CCCTC-binding factor activates PARP-1 affecting DNA methylation machinery. J Biol Chem 283, 21873–21880 (2008). 18539602 10.1074/jbc.M801170200 PMC2494936

[b62] Zampieri M. *et al.* ADP-ribose polymers localized on Ctcf-Parp1-Dnmt1 complex prevent methylation of Ctcf target sites. Biochem J 441, 645–652 (2012). 21985173 10.1042/BJ20111417 PMC3258657

[b63] Davalos-Salas M. *et al.* Gain of DNA methylation is enhanced in the absence of CTCF at the human retinoblastoma gene promoter. BMC Cancer 11, 232 (2011). 21663659 10.1186/1471-2407-11-232 PMC3145615

[b64] Vetchinova A. S., Akopov S. B., Chernov I. P., Nikolaev L. G. & Sverdlov E. D. Two-dimensional electrophoretic mobility shift assay: identification and mapping of transcription factor CTCF target sequences within an FXYD5-COX7A1 region of human chromosome 19. Anal Biochem 354, 85–93 (2006). 16701069 10.1016/j.ab.2006.03.052

[b65] Livak K. J. & Schmittgen T. D. Analysis of relative gene expression data using real-time quantitative PCR and the 2(-Delta Delta C(T)) Method. Methods 25, 402–408 (2001). 11846609 10.1006/meth.2001.1262

