# Corrigendum: Epigenetic silencing of the XAF1 gene is mediated by the loss of CTCF binding

**DOI:** 10.1038/srep20462

**Published:** 2016-02-08

**Authors:** Georgina Victoria-Acosta, Karla Vazquez-Santillan, Luis Jimenez-Hernandez, Laura Muñoz-Galindo, Vilma Maldonado, Gustavo Ulises Martinez-Ruiz, Jorge Melendez-Zajgla

Scientific Reports 5: Article number: 1483810.1038/srep14838; published online: 10072015; updated: 02082016.

In the Supplementary Information file originally published with this Article, Supplementary Figures 1c and 1d were incorrectly labelled as Figure 1a and 1b. In addition, Supplementary Figures 2b, 2c and 2d were incorrectly labelled as Figure 1c, 1d and 1e. Lastly, Supplementary Figures 1a, 1b and 2a were omitted. These errors have been corrected in the Supplementary Information that now accompanies the Article.

