# Supplementary material for: Epigenetic silencing of the XAF1 gene is mediated by the loss of CTCF binding
Source: Sci Rep. 2015 Oct 7;5:14838. doi: 10.1038/srep14838 (PMC4595840; doi:10.1038/srep14838)
Supplement: Supplementary Figures [file srep14838-s1.pdf]

Epigenetic silencing of the XAF1 gene is mediated by the loss of CTCF binding.

Georgina Victoria-Acosta, Gustavo U. Martinez-Ruiz, Karla Vazquez-Santillan, Luis Jimenez-Hernandez, Laura Muñoz-Galindo, V Maldonado, Jorge Melendez-Zajgla

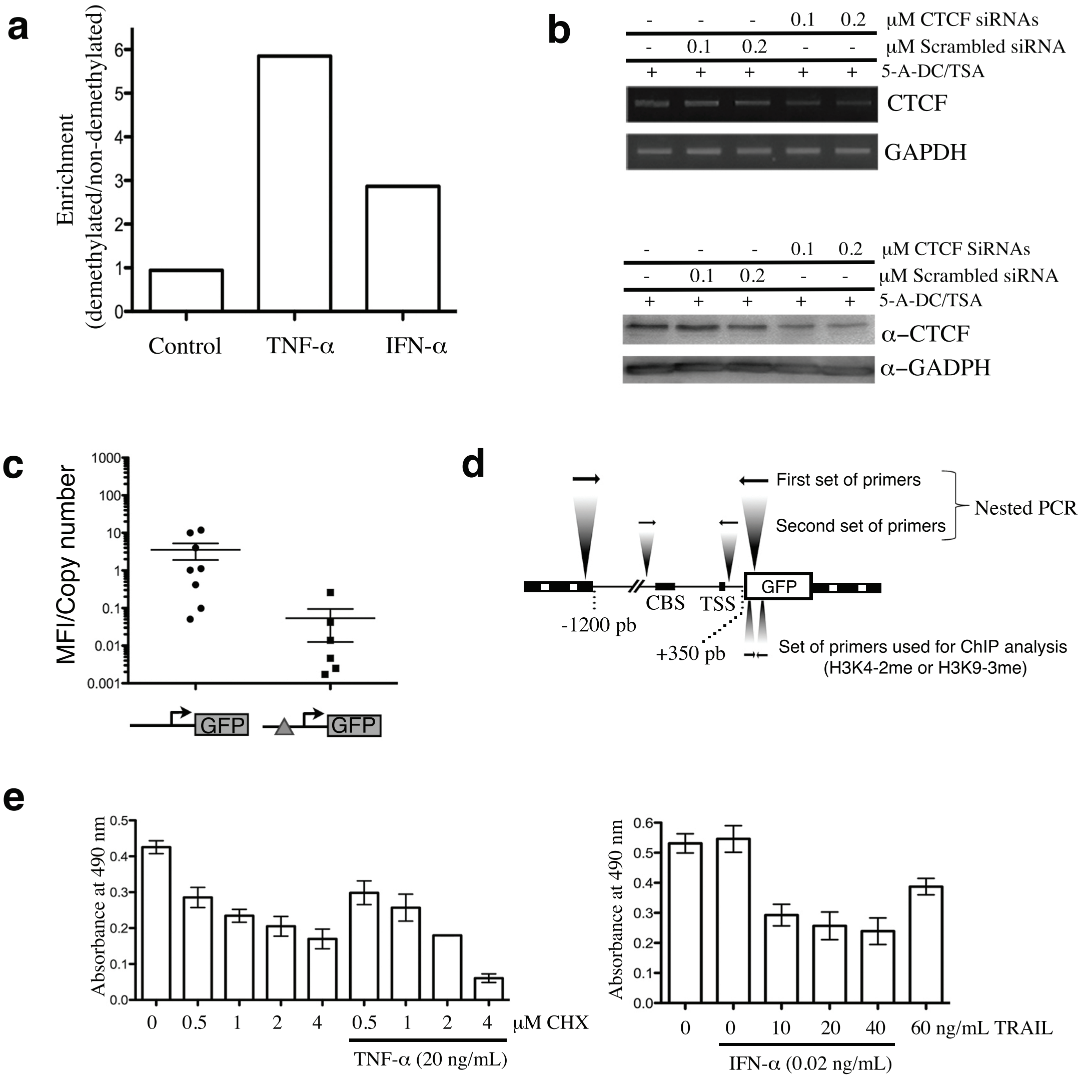

SUPPLEMENTARY FIGURE LEGENDS

Supplementary Figure 1. (A) ACHN cells were pre-treated with *5-Aza-2′*-deoxycytidine (5-A-DC) at 5 μM and Trichostatin-A (TSA) at 0.2 μM for 3 days before stimulation with either TNF-α (20 ng/mL) or IFN-α (0.02 ng/mL). ACHN cells without pre-treatment with demethylating agents (non-demethylated cells) were also stimulated with either TNF-α or IFN-α. After, immunoprecipitation assays were performed using a specific antibody against CTCF protein. DNA recovered after ChIP assays was subjected to quantitative PCR amplification using specific primers to amplify the CTCF binding site in the XAF1 promoter. (B) MCF-7 cells were pre-treated as indicated in (A). After, the cells were transiently transfected with CTCF siRNAs or control scramble siRNAs at the indicated concentrations. At 48 h post-transfection, total RNA and total protein were isolated. RT-PCR (upper panel) and Western blotting (lower panel) were used to analyse the expression of both the XAF1 and CTCF genes. (C) The Median Fluorescence Intensity (MFI) from each single-cell clone containing either peGFPN1-XAF1 or peGFPN1-Δ-CTCF-XAF1 constructs was normalized against its copy number, which was measured by quantitative PCR. (D) A schematic representation is shown of the localization of primers used in PCR amplification using bisulphite-modified DNA and in ChIP assays against either H3K4-2me or H3K9-3me. (E) MCF-7 cells were pre-treated as indicated in (A). After, the cells were stimulated with cycloheximide (CHX) alone or in combination with TNF-α (left graph). MCF-7 cells previously pre-treated with demethylating agents were stimulated with TRAIL alone or in the presence of IFN-α (right graph). The median with the range was plotted from two independent experiments. CTCF-binding site (CBS); Transcription start site (TSS).
